# Identifying and Validating of an Autophagy-Related Gene Signature for the Prediction of Early Relapse in Breast Cancer

**DOI:** 10.3389/fendo.2022.824362

**Published:** 2022-02-16

**Authors:** Yu Min, Yang Feng, Haojun Luo, Daixing Hu, Xiaoyuan Wei, Danshuang He, Guobing Yin, Shenghao Fan

**Affiliations:** ^1^ Department of Breast and Thyroid Surgery, The Second Affiliated Hospital of Chongqing Medical University, Chongqing, China; ^2^ Department of Cardiology, The Second Affiliated Hospital, Chongqing Medical University, Chongqing, China

**Keywords:** breast cancer, early relapse, autophagy, signature, nomogram

## Abstract

**Background:**

Compelling evidence has demonstrated the pivotal role of autophagy in the prognosis of breast cancer. Breast cancer (BC) patients with early relapse consistently exhibited worse survival.

**Methods:**

The autophagy-related genes were derived from the Human Autophagy Database (HADb) and high-sequencing data were obtained from The Cancer Genome Atlas (TCGA). Discrepantly expressed autophagy genes (DEAGs) between early relapse and long-term survival groups were performed using the Linear Models for Microarray data (LIMMA) method. Lasso Cox regression analysis was conducted for the selection of the 4-gene autophagy-related gene signature. GSE42568 and GSE21653 databases were enrolled in this study for the external validation of the signature. Then patients were divided into high and low-risk groups based on the specific score formula. GSEA was used to discover the related signaling pathway. The Kaplan-Meier curves and the receiver operating characteristic (ROC) curves were used to evaluate the discrimination and accuracy of the 4-gene signature.

**Results:**

A signature composed of four autophagy-related mRNA including APOL1, HSPA8, SIRT1, and TP73, was identified as significantly associated with the early relapse in BC patients. Time-dependent receiver-operating characteristic at 1 year suggested remarkable accuracy of the signature [area under the curve (AUC = 0.748)]. The risk score model based on the autophagy-related signature showed favorable predicting value in 1-, 2-, and 3-year relapse-free survival (RFS) in training and two validating cohorts. The GSEA displayed gene sets were remarkably enriched in carcinogenic activation pathways and autophagy-related pathways. The nomogram involving three variables (progesterone receptor status, T stage, and 4-gene signature) exhibited relatively good discrimination with a C-index of 0.766.

**Conclusions:**

Our study establishes an autophagy-related 4-gene signature that can effectively stratify the high-risk and low-risk BC patients for early relapse. Combined with the clinicopathological variables, the signature could significantly help oncologists tailor more efficient treatment strategies for BC patients.

## Introduction

Breast cancer (BC) is currently the most frequent malignancy and one of the leading causes of cancer death in the United States (estimated 279,100 new cases and 42,690 death) (1) and China mainland (estimated 304,000 new cases and 70,000 death) (2). Although the long-term survival of patients with BC has been significantly increased in the past years with the application of targeted therapy (3), endocrine therapy (4), and even immunotherapy (5, 6), early relapse (2 years after initial treatment) with metastasis could reverse this favorable outcome (7, 8). Regardless of the prognosis, all women with BC are at risk for early recurrence. According to a recent review report, nearly 50% of early recurrences occur within 5 years of surgery, and they peak at 2 years after surgery in women treated with adjuvant tamoxifen (9). Besides, early relapse in BC patients is frequently associated with poor clinicopathological features [such as young age (10), late TNM stage, poor differentiation grade, and worse histopathological type (11, 12)] and resistance to adjuvant chemotherapy or endocrine therapy (13–16). Those cases who developed early relapse consistently tended to have poorer long-term survival rates. Notably, a recent study has demonstrated that BC patients experienced altered hormone receptor and HER2 status throughout tumor progression, which significantly influences survival (17). Thus, for the great heterogeneity of BC, the prognosis varies significantly in BC patients with the same stage and comparable clinicopathological features. For this reason, hall markers and other biological indicators could help to predict the recurrence of BC (18).

Autophagy is a routine physiological process associated with aging and human disease *via* guiding the degradation of damaged, denatured, or senescent proteins and organelles in lysosomes (19, 20). Accordingly, compelling evidence has demonstrated that autophagy plays a pivotal role in tumor growth, metastasis, and recurrence of BC, which could maintain the homeostasis and the survival of BC cells by removing dysfunctional or unnecessary substances (21–24). On the other hand, accumulating evidence showed that autophagy-related genes were significantly involved in the regulations of the autophagy process. For instance, recent two basic research demonstrated that MTA1 (metastasis-associated 1) (13) and long noncoding RNA H19 (14) were the regulators of autophagy in resistance to the endocrine therapy (tamoxifen). However, Marsh et al. (25) discovered autophagy could inhibit the metastasis of BC cells by accumulating the autophagy cargo receptor (ACR), neighbor to BRCA1. A number of coding RNA (mRNA) and non-coding RNAs (microRNA, lncRNA, and circRNA) signatures have been identified for predicting the proliferation and prognosis of BC patients (26–30). Nonetheless, most of these signatures focused on overall survival, there is still a lack of work on investigating the impact of mRNA on the relapse-free survival of BC, and none of the previous studies have concentrated on early relapse. Therefore, identifying autophagy-related mRNA signature could not only easily help oncologists classify the BC patients with a high risk of early relapse but also make more efficient therapeutic modalities at an earlier stage of a patient’s treatment (31).

In the present study, we conducted an autophagy-related 4-gene signature to predict the early relapse of BC patients and construct a nomogram for predicting the 1-, 2-, and 3-year RFS probability during clinical practice.

## Materials and Methods

### Data Source Collection

**Figure 1:**
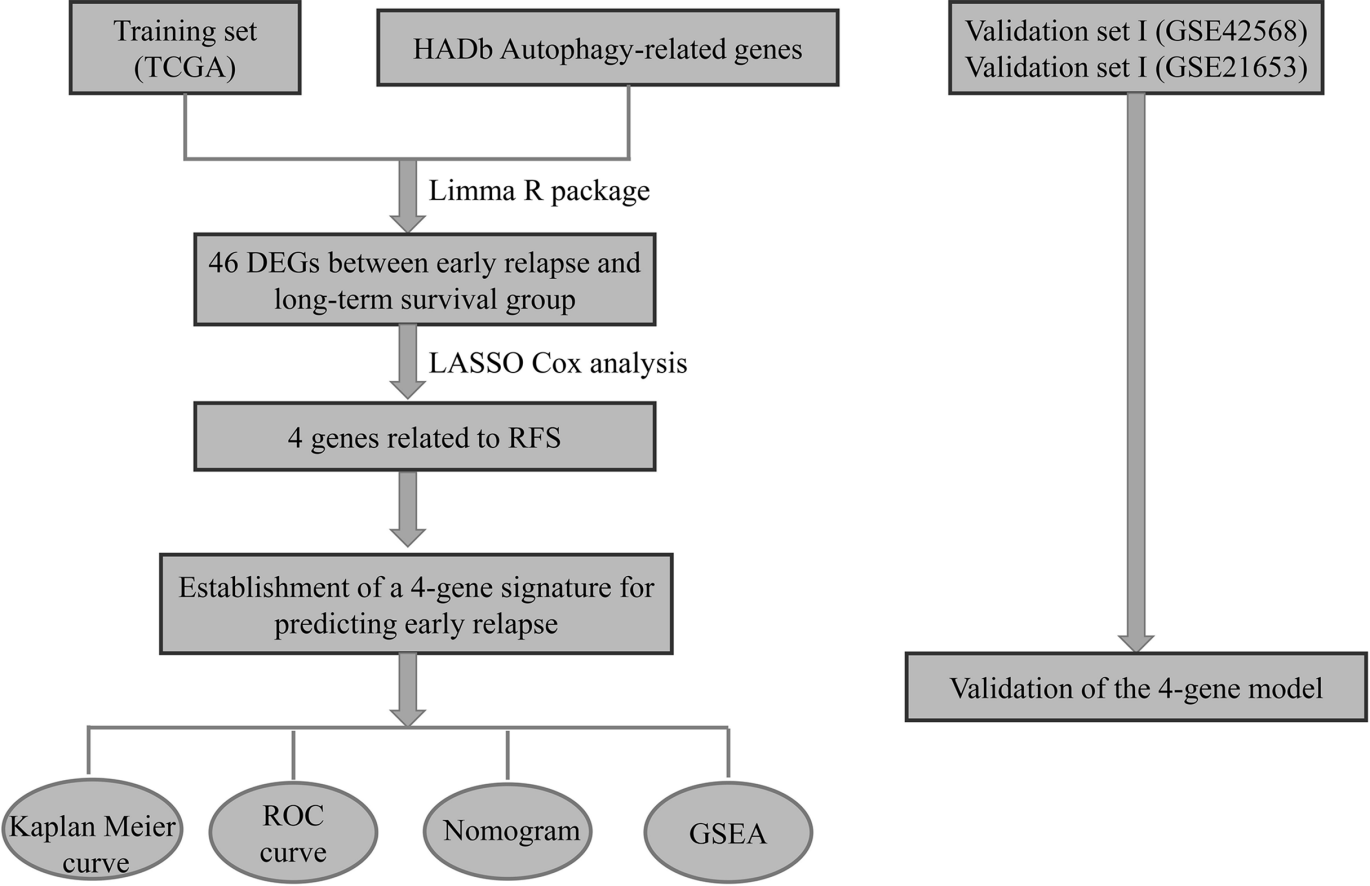
The autophagy-related 4-gene signature selection and validation process.

The messenger RNA (mRNA)-seq expression and clinicopathological characteristics of 1,025 BC patients were obtained from the TCGA program website (https://cancergenome.nih.gov/). Meanwhile, the autophagy-related genes were derived from the human autophagy database (HADb, http://www.autophagy.lu/). After excluding BC patients with incomplete clinicopathological medical records and patients initially diagnosed with metastasis, there were 785 BC patients included for further analysis. The data from the TCGA database were assigned as a training cohort (**Figure 1**). Moreover, two Gene Expression Omnibus (GEO) cohorts including the GSE42568 and GSE21653 datasets (detailed clinical information was summarized in **Table S1**) were obtained from the GEO database (https://www.ncbi.nlm.nih.gov/geo/) and assigned as the validation cohorts. All GEO datasets were produced by the Affymetrix HG-U133 plus 2.0. Raw microarray was normalized using Robust Multichip Average (32). When multiple probes were mapped to the same Entrez Gene ID, we used the mean value to represent its average expression level.

### Ethics Approval

The protocol for this study was approved by Chongqing Medical University. Ethical approval was waived by the local Ethics Committee of the Chongqing Medical University in view of the retrospective nature of the study and all the procedures being performed were part of the routine care.

### Identification of Autophagy-Related mRNA Signature for the Early Relapse of BC

Recurrence of BC patients was frequently occurred within 5 years after the initial treatment, while the first 2 years were the peak of recurrence (9). However, the definition of early relapse in BC patients is still ambiguous in recently published literature (33–36). Thus, early relapse in the present study was defined as the locoregional recurrence or distant metastasis within a short-term of 2 years follow-up after the initial primary resection. Samples in the training set were selected and divided into early relapse group and long-term survival group (no relapse at least 5 years follow-up). The calculations of differentially expressed genes (DEGs) between early relapse and long-term survival BC patients were conducted using the linear models for microarray data (LIMMA) method. The threshold for identification of DEGs was set as P value < 0.1. Besides, the LASSO Cox regression model (37) was used to select the most significantly relapse-associated mRNA of all the DEGs. A risk score model containing both coefficients and mRNA expression levels was established to generate the risk score for all BC patients in the training cohort. Based on the risk score, patients were divided into high-risk and low-risk groups with the median risk score as the cut-off point.

### Gene Set Enrichment Analysis

The Gene Set Enrichment Analysis (GSEA, http://www.broadinstitute.org/gsea/index.jsp) was applied to evaluate differences between the low-risk and high-risk groups. Namely, Gene Ontology (GO) enrichment analysis and Kyoto Encyclopedia of Genes and Genomes (KEGG) pathway analysis were conducted to differentially expressed genes between these two groups. Normal P values <0.05 were regarded as statistically significantly enriched.

### Validation Analysis

To further confirm the classification reliability and prognosis value of the 4-gene signature analyzed by TCGA, similar analyses were performed on GSE42568 and GSE216533 datasets to validate the prognostic significance of this autophagy-related signature.

### Statistical Analysis

Survival differences between the low-risk and high-risk groups in each set were assessed by the Kaplan–Meier estimate and compared *via* the log-rank test. Baseline characteristics between low-risk and high-risk groups in each set were compared using the Pearson-chi square test (minimal expected value > 5). Multivariate Cox regression analysis and data stratification analysis were exploited to evaluate the independent prognostic significance of risk score and clinicopathological factors in predicting the RFS of BC patients. Time-dependent receiver-operating characteristic (ROC) analysis was used to investigate the prognostic and predictive accuracy of the signature. To access the probability of RFS survival in BC patients, a nomogram was subsequently developed based on the risk score and clinical features by using the “rms” R package. And the predictive feasibility of the nomogram was weighed by the Harrell concordance indexes (C-index) and calibration curves. All statistical analyses were performed with the use of R (version 4.0.3, www.r-project.org). All statistical tests were two-tailed, and P values < 0.05 were considered statistically significant.

## Results

### Identification of an Autophagy-Related Gene Signature for the Early Relapse of BC

**Figure 2:**
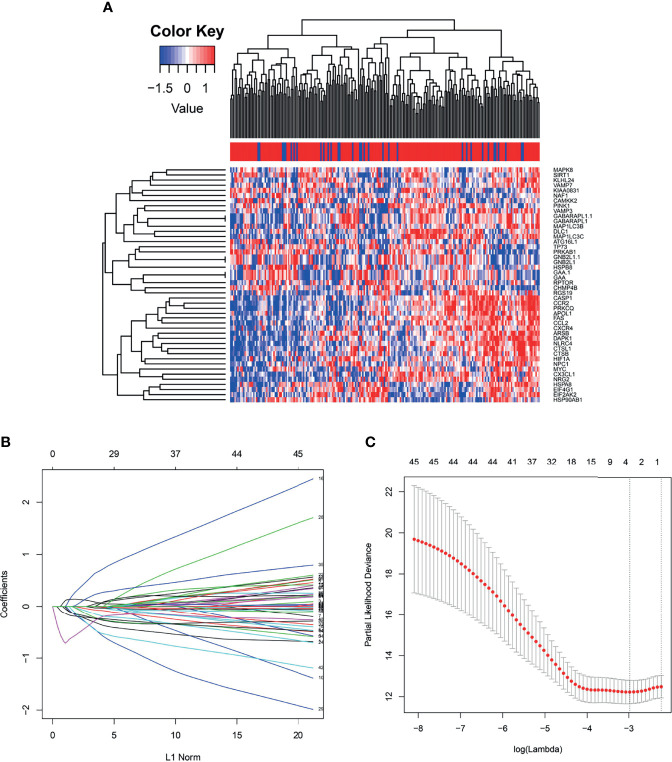
**(A)** The heat map demonstrates forty-six differentially expressed mRNA in breast cancer among early relapse and long-term survival group both in training cohort; **(B)** LASSO coefficient profiles of the 4 early relapse-associated mRNA. A vertical line is drawn at the value chosen by 10-fold cross-validation; **(C)** X-tile analysis of the 4 selected GRGs.

Generally, we take the intersection of mRNA from the TCGA database with 222 autophagy genes in HADb. 46 differentially expressed autophagy-related genes were identified between the early relapse group and long-term survival group by using the “limma” package in the R software. These genes were subsequently included for LASSO analysis (**Figure 2**). Based on the LASSO analysis, four genes including the APOL1, HSPA8, SIRT1, and TP73, were regarded as the independent prognostic factor in early relapse BC patients. A gene-based prognostic model was further established to evaluate the RFS risk for each patient. The results are as follows: Risk score= (-0.209* status of APOL1) +(0.387* status of HSPA8) +(-1.073* status of SIRT1) +(-0.233* status of TP73). Thus, BC patients were divided into high-risk and low-risk groups with the median risk score as the cut-off point (**Table 1**).

**Table 1:** The demographic characteristics of breast cancer patients among high-risk and low-risk groups according the autophagy-related 4-gene signature.

Variables	Subgroup	No. (%) of patients			
		Total (n=785)	High risk (n=392)	Low risk (n=393)	^a^ *P*
**Literality**	**Left**	424 (54.0)	208 (53.1)	216 (55.0)	0.593
	**Right**	361 (46.0)	184 (46.9)	177 (45.0)
**Age**	**<50**	221 (28.2)	123 (31.4)	98 (24.9)	**0.045**
	**≥50**	564 (71.8)	269 (68.6)	295 (75.1)
**ER**	**Positive**	562 (71.6)	238 (60.7)	324 (82.4)	**<0.001**
	**Negative**	185 (23.6)	130 (33.2)	55 (14.0)	
	**Other**	38 (4.8)	24 (6.1)	14 (3.6)	
**PR**	**Positive**	496 (63.2)	205 (52.3)	291 (74.0)	**<0.001**
	**Negative**	248 (31.6)	162 (41.3)	86 (21.9)	
	**Other**	41 (5.2)	25 (6.4)	16 (4.1)	
**HER2**	**Positive**	117 (14.9)	68 (17.3)	49 (12.5)	0.350
	**Negative**	414 (52.7)	199 (50.8)	215 (54.7)	
	**Other**	254 (32.4)	125 (31.9)	129 (32.8)	
**pT**	**T1**	214 (27.3)	105 (26.8)	109 (27.7)	**0.003**
	**T2**	467 (59.5)	249 (63.5)	218 (55.5)	
	**T3**	85 (10.8)	27 (6.9)	58 (14.8)	
	**T4**	19 (2.4)	11 (2.8)	8 (2.0)	
**pN**	**N0**	399 (50.8)	201 (51.3)	198 (50.4)	0.333
	**N1**	256 (32.6)	125 (31.9)	131 (33.3)	
	**N2**	87 (11.1)	46 (11.7)	41 (10.4)	
	**N3**	43 (5.5)	20 (5.1)	23 (5.9)	
**Surgery**	**Lumpectomy**	178 (22.7)	105 (26.8)	73 (18.6)	**0.010**
	**Mastectomy**	359 (45.7)	162 (41.3)	197 (50.1)	
	**Other**	248 (31.6)	125 (31.9)	123 (31.3)	

ER, Estrogen receptor; PR, Progesterone receptor; HER-2, Human epidermal growth factor receptor-2; pT, pathologically diagnosed tumor size; pN, pathologically diagnosed lymph node status.

a Pearson’s Chi-squared test.

Bold values indicate statistical significance (p<0.05).

### The Prognostic Value of 4-Gene Signature in Training and Validating Cohorts

**Figure 3:**
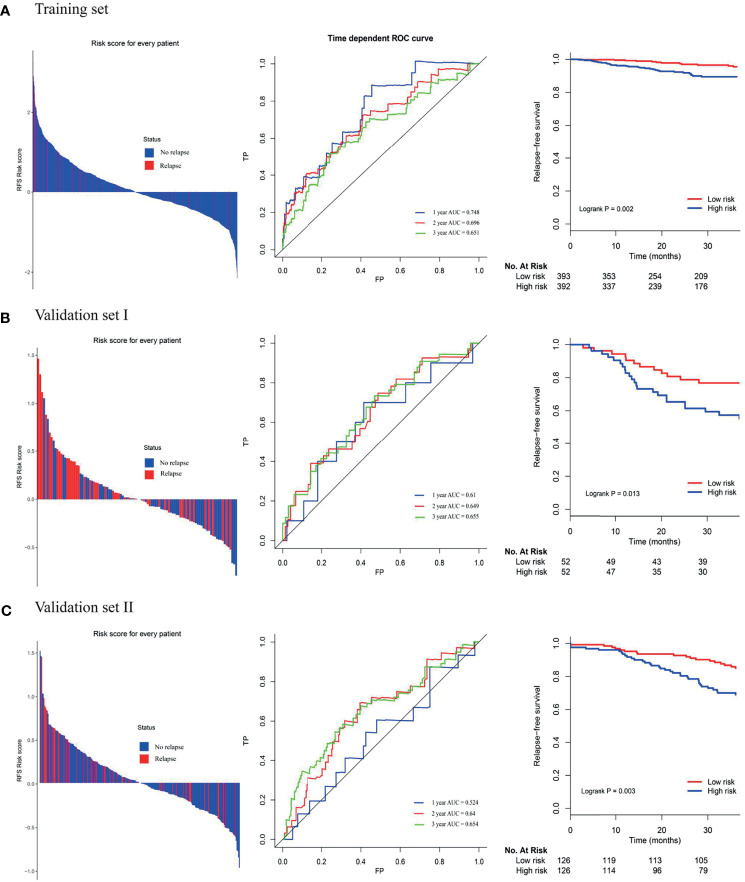
**(A)** Distribution of risk score, time-dependent ROC curves at 1, 2, and 3 years and Kaplan–Meier survival analysis between patients at low and high risks of relapse in training cohort; **(B)** first external validation cohort; **(C)** second external validation cohort.

Among the high-risk group and low-risk group, the distribution of risk score and relapse status of BC patients were displayed. The results showed that the higher the risk score, the higher the morbidity rate was observed in the training group and two validating groups (**Figure 3**). Similarly, the Kaplan-Meier survival analysis demonstrated that the RFS of the high-risk group was significantly inferior to those of the low-risk group in the training group and two validating groups (log-rank p=0.002 in the training set, log-rank p=0.013 in the validation set I, log-rank p=0.003 in the validation set II, respectively). Moreover, the time-dependent ROC analyses at 1-, 2-, and 3-year were also conducted to evaluate the accuracy of the 4-gene classifier. In the training cohort, the AUC of 1-, 2-, and 3-year was 0.748, 0.696, and 0.651, respectively. Accordingly, a relatively promising AUC value was also observed in the validating sets (validating set I: the AUC of 1-, 2-, and 3-year was 0.611, 0.649, and 0.655, respectively; validating set II: the AUC of 1-, 2-, and 3-year was 0.524, 0.640, and 0.654, respectively).

### Establishment of a Predictive Nomogram

**Figure 4:**
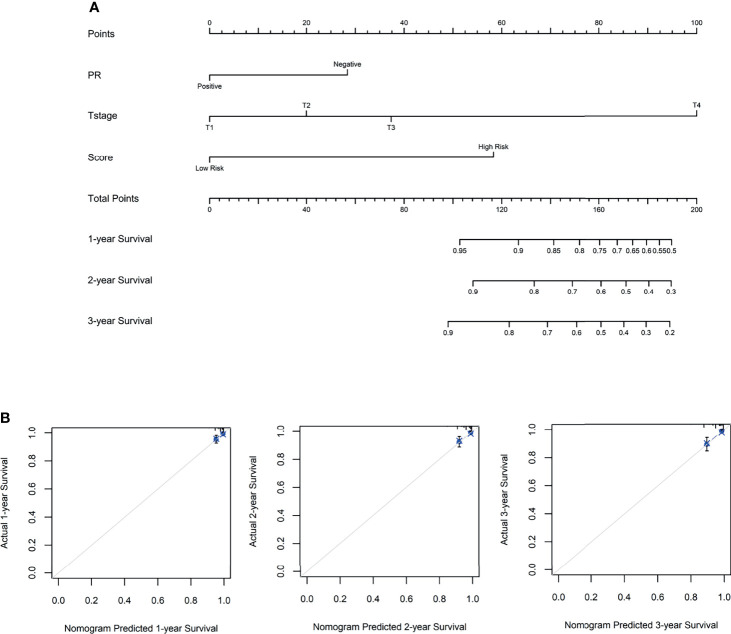
**(A)** The nomogram for predicting the 1-, 2-, and 3- year relapse-free survival in breast cancer patients, based on the autophagy-related 4-gene signature selection and clinical factors. **(B)** The 1-, 2- and 3-year calibration curves were derived from the nomogram, respectively.

To access the independence and accuracy of the 4-gene signature in predicting the RFS of BC patients. The univariate and multivariate Cox analyses integrated with the clinicopathological characteristics were performed (**Table 2**). At univariate analysis, PR status, primary tumor size, regional lymph node status, and the 4-gene based signature were significantly associated with the early relapse of BC patients. During the multivariate analysis, larger tumor size (T2: HR=1.82, 95%CI: 0.51- 6.38, T3: HR=3.06, 95%CI: 0.50- 18.61, T4: HR=19.99, 95%CI: 3.88- 102.76, p=0.001) and high-risk BC patients derived from the 4-gene classifier (HR=5.73, 95%CI: 1.63- 20.16, p=0.006) were identified as the independent risk factors in promoting the early relapse of BC. PR status reached marginal significance (HR=2.34, 95%CI: 0.96- 5.73, p=0.063). A novel nomogram (**Figure 4**) was subsequently established with the three variables involvement (PR status, tumor size, and 4-gene signature). Optimally, the model contained a satisfying C-index of 0.766 (95%CI: 0.604-0.927). Moreover, three calibration curves for evaluating the accuracy of the predictive ability in short-term RFS were also performed *via* 1000 bootstrap repetitions (**Figure 4**). The curves (apparent, ideal, and bias-corrected lines) suggested a promising agreement in the training model.

**Table 2:** The univariate and multivariate Cox regression analysis in the early relapse of breast cancer in the training group.

Variables	Subgroup	Univariable		Multivariable	
		Hazard ratio	*P*	Hazard ratio	*P*
**Laterality**	**Left**	1	0.285	/	
	**Right**	1.640 (0.662, 4.064)			
**Age**	**<50**	1	0.175	/	
	**≥50**	0.550 (0.231, 1.305)			
**ER**	**Positive**	1	0.041	1	0.469
	**Negative**	1.569 (1.018, 2.418)		0.643 (0.194, 2.125)	
**PR**	**Positive**	1	**0.006**	1	0.063
	**Negative**	1.856 (1.195, 2.883)		2.340 (0.955, 5.732)	
**HER2**	**Positive**	1	0.396	/	
	**Negative**	0.814 (0.507, 1.308)			
**pT**	**T1**	1	**0.001**	1	**0.001**
	**T2**	1.991 (0.567, 6.987)		1.820 (0.518, 6.389)	
	**T3**	1.818 (0.304, 10.881)		3.061 (0.503, 18.619)	
	**T4**	19.395 (3.876, 94.048)		19.992 (3.889, 102.767)	
**pN**	**N0**	1	**0.025**	1	0.192
	**N1**	0.357 (0.101, 1.265)		0.349 (0.097, 1.259)	
	**N2**	0.810 (0.181, 3.620)		0.854 (0.184, 3.956)	
	**N3**	3.477 (1.118, 10.816)		2.051 (0.568, 7.415)	
**Surgery**	**Lumpetomy**	1	0.762	/	
	**Mastectomy**	0.896 (0.306,2.621)			
	**Other**	0.660 (0.201, 2.162)			
**Score**	**Low risk**	1	**0.003**	1	**0.006**
	**High risk**	6.304 (1.857, 21.407)		5.737 (1.632, 20.167)	

ER, Estrogen receptor; PR, Progesterone receptor; HER-2, Human epidermal growth factor receptor-2; pT, pathologically diagnosed tumor size; pN, pathologically diagnosed lymph node status.

Bold values indicate statistical significance (p<0.05).

### Gene Set Enrichment Analysis

**Figure 5:**
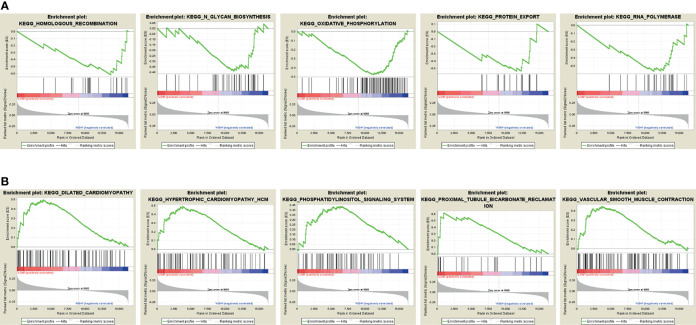
Gene Set Enrichment Analysis (GSEA). **(A)** GSEA shows a significant enrichment of cancer-related pathways in the high-risk group based on the training cohort. **(B)** GSEA shows a significant enrichment of cancer-related pathways in the low-risk group based on the training cohort.

The KEGG pathway analysis was conducted to discover the associated biological signaling pathway of 4 autophagy-related mRNA sets. Notably, differentially expressed genes between high-risk and low-risk groups were determined. Namely, the GSEA results indicated that the genes enriched in the high-risk group were related to the regulation of homologous recombination, N-glycan biosynthesis, oxidative phosphorylation, protein export, and RNA polymerase (**Figure 5**). On the contrary, in the low-risk group, the autophagy-related gene sets were involved in pathways related to dilated cardiomyopathy, cardiomyopathy HCM, phosphatidylinositol signaling system, proximal tubule bicarbonate reclamation, and vascular smooth muscle contraction (**Figure 5**).

## Discussion

To date, BC has become the leading malignancy among women worldwide (1, 2, 38) with a promising relatively higher 5- year survival rate, compared with other invasive cancers. Nevertheless, survivors can experience early recurrence with resistance to the initial treatments paralleled by highly invasive metastasis (9, 35). TNM stage and immunohistochemical indicators like ER, PR, Her-2, and Ki-67 index were frequently used to access the prognosis of BC patients. Chen et al. (12) determined that the late-stage (p< 0.001), poor differentiated grade (p = 0.002), PR-negative status (p = 0.014), and HER2-negative status (p = 0.033) were significant associated with the early relapse of BC. However, Huang et al. (11) determined that the cancer TNM stage was significantly associated with the early-relapse in BC patients, while clinical variables including age, tumor location, ER status, PR status, or HER2 status were not. In addition, a different survival pattern has been observed in BC patients with a relatively similar condition during clinical practice. These results indicated that genetic biomarkers also played a pivotal role in regulating tumor cell cycle progression and metastasis.

Regarding the gene signatures, previous works highlighted that the imbalance of cell proliferation and apoptosis, as well as autophagy regulation disorder, might also be attributed to the occurrence and development of BC. Namely, autophagy is a pivotal process in control of cell fate and significantly correlates with apoptosis *via* inactivating the mammalian target of rapamycin (mTOR) signaling pathway or directly activates the initiation step of autophagy by phosphorylating unc-51-like autophagy activating kinase 1 (ULK1). In terms of cancer initiation, autophagy is considered tumor-suppressive due to its cytoprotective role (23, 25, 39, 40). Notably, Marsh et al. (25) discovered autophagy could inhibit the metastasis of BC cells by accumulating the autophagy cargo receptor (ACR), neighbor to BRCA1. Moreover, recent two basic research demonstrated that MTA1 (metastasis-associated 1) (13) and long noncoding RNA H19 (14) were the regulators of autophagy in resistance to the endocrine therapy (tamoxifen). On the contrary, several studies (22, 41) demonstrated the autophagy was positively associated with the tumor growth, metastasis, and recurrence of BC, which could maintain the homeostasis and the survival of BC cells by removing dysfunctional or unnecessary substances. Therefore, autophagy is a powerful but double-edged sword, which had an essential impact on the prognosis of BC (41).

In the present study, 4 autophagy-related mRNA including APOL1, HSPA8, SIRT1, and TP73 were pivotal genes in the RFS of BC. Of these genes, APOL1 (apolipoprotein-L1) has been observed significantly associated with kidney disease, especially in terms of HIV-related chronic renal disease (42, 43). In regulating the proliferation and metastasis of cancer cells, recent studies speculated the phenotype of APOLs was involved in several cancers’ metastasis *via* the strong reduction of cellular adherence and increased in cell motility, together with an important reduction of the capacity for apoptosis (44–47). Besides, members of the heat-shock protein 70 (HSPA) family gained plenty of attention as a potential target for tumor therapy, which could promote cancer cell growth by different mechanisms (48–50). For instance, Rohde et al. (50) demonstrated the suppression role of HSPA in “HeLa” cells, namely, depletion of HSPA and HSPA2 arrested cancer cells in G2/M and G1, respectively. Regarding the SIRT1 (Sirtuin-1) mRNA, it significantly participated in gene regulation, genome stability maintenance, apoptosis, autophagy, and tumorigenesis (51). As recent studies reported, a downregulation of SIRT1 has already been described in gastric cancer (52) and breast cancer (53, 54). Zhang et al. demonstrated the activation of SIRT1 could suppress gastric cancer cells proliferation and metastasis *via* STAT3/MMP-13 signaling pathway (52). Meanwhile. Latifkar et al. (54) reported that inhibition of SIRT1 would impair the lysosomal function, resulting in the enhanced secretion of pro-tumorigenic exosomes which might reconstruct the extracellular matrix and enhance the invasive properties of cultured BC cells. Additionally, accumulating evidence has proved the dysfunction of TP73 (tumor protein p73) was associated with the proliferation and prognosis of different cancers (55). Notably, *in vitro* study, Sharif et al. (56) demonstrated that high expression levels of TP73 suppressed the proliferation of BC *via* enhanced autophagy and cell death. Alternatively, knockdown of TP73 decreased NAMPT (nicotinamide phosphoribosyl transferase) inhibition-induced autophagy and cell death.

Regarding the clinicopathological characteristics of BC patients, only primary tumor size was significantly associated with the early relapse of BC patients after stepwise multivariate analysis (adjust p=0.001). Interestingly, negative progesterone receptor status trended towards significantly increasing the risk of early relapse of BC patients (adjust p=0.063). Previously, compare to the prognostic value of estrogen receptor and HER2 status, progesterone receptor status was not so important. However, recent studies have demonstrated that progesterone receptor-negative tumors have generally been shown to have a poorer prognosis than progesterone receptor-positive tumors (57). Notably, evidence from one large population-based study, negative progesterone receptor status was associated with higher differentiation grade and subsequent recurrence score (58). Meanwhile, Zhang et al. demonstrated that the expression levels of progesterone receptor (cutoff point: 55%) played a pivotal role in predicting the relapse of hormone receptor-positive BC patients (59). However, the underlying mechanism and potential signaling pathway are still not clear but worth further investigation.

To our knowledge, we first discovered this autophagy-related 4-gene signature involved in the early relapse of BC. Based on the 4-gene signature, a risk score model was successfully established. And it was externally validated by two cohorts of GSE42568 and GSE21653, suggesting the favorable reproducibility of this signature in BC. However, the underlying molecular mechanism and signaling pathways of this signature are still inadequately clarified in BC. Nonetheless, the GSEA showed that the genes enriched in the high-risk group were related to the regulation of homologous recombination, N-glycan biosynthesis, oxidative phosphorylation, protein export, and RNA polymerase cancer-related signaling conduction. Alternatively, among the low-risk population, the autophagy-related gene sets were involved in pathways related to dilated cardiomyopathy, cardiomyopathy HCM, phosphatidylinositol signaling system, proximal tubule bicarbonate reclamation, and vascular smooth muscle contraction. Thus, further investigation of the underlying mechanisms may be meaningful. Additionally, constructing a convenient while reliable autophagy-related mRNA signature for identifying the risk biomarkers in promoting early relapse of BC would make up for the deficiency of clinicopathological classification, and further assist oncologists in formulating more efficient treatment modalities at an earlier stage of patients’ management. For this reason, we constructed a nomogram combined with two clinicopathological prognostic factors to predict the 1-, 2-, and 3-year RFS of BC patients in an effective quantitative approach. An optimal C-index of 0.766 was achieved which indicated the feasibility of identifying the high-risk BC patients with early relapse during clinical practice.

Indeed, there are some limitations in the current study needed to be mentioned and addressed in future works. First, this is a retrospective-designed study, and all BC samples were identified from the public database which inevitably weakened the findings we determined. Second, further basic research in our department and other medical centers is merited to external validate our conclusions and elucidate the functional roles of autophagy-related mRNA signature involved in the early relapse of BC. Moreover, with a significant improvement of overall survival in BC patients, longer follow-up (like 10 years) time could better help oncologists predict the clinical outcome in these patients. Last, the risk score model and nomogram can only be applied to predict early relapse in BC patients, and its prognostic role in the different molecular subtypes of BC warrants further evaluation.

## Conclusion

In summary, our works demonstrate that 4 autophagy-related mRNA (APOL1, HSPA8, SIRT1, and TP73) are significantly associated with the early relapse of breast cancer during the postoperative follow-up. Based on the autophagy-related mRNA signature risk score classifier, good discrimination in identifying the BC patients with a high risk of early relapse is achieved. Moreover, we successfully establish and validate a utility nomogram derived from the risk scores combining tumor size and PR status for clinically predicting the 1-, 2-,3-year RFS probability in BC patients after initial surgical intervention. Future prospective clinical trials could verify the clinical significance of our autophagy-related mRNA signature in stratifying early relapse in BC patients postoperatively. The mechanisms and underlying signaling of the identified genes on the early relapse of BC are also needed to be further explored.

## Author’s Note

The software application generated during and/or analyzed during the current study are available from the corresponding author on reasonable request.

## Data Availability Statement

The datasets presented in this study can be found in online repositories. The names of the repository/repositories and accession number(s) can be found in the article/**Supplementary Material**.

## Author Contributions

(I) Conception and design: YM and YF. (II) Administrative support: SF and GY. (III) Provision of study materials or patients: YF, XW, and YM. (IV) Collection and assembly of data: YM and HL. (V) Data analysis and interpretation: YM and XW. (VI) Manuscript writing: All authors. All authors contributed to the article and approved the submitted version.

## Conflict of Interest

The authors declare that the research was conducted in the absence of any commercial or financial relationships that could be construed as a potential conflict of interest.

## Publisher’s Note

All claims expressed in this article are solely those of the authors and do not necessarily represent those of their affiliated organizations, or those of the publisher, the editors and the reviewers. Any product that may be evaluated in this article, or claim that may be made by its manufacturer, is not guaranteed or endorsed by the publisher.
