# Supplementary material for: Identifying and Validating of an Autophagy-Related Gene Signature for the Prediction of Early Relapse in Breast Cancer
Source: Front Endocrinol (Lausanne). 2022 Feb 16;13:824362. doi: 10.3389/fendo.2022.824362 (PMC8888901; doi:10.3389/fendo.2022.824362)
Supplement: Supplementary file 1 [file Table_1.docx]

| **Variables** | **Subgroup** | **GSE42568 (n=104)** | **GSE21653 (n=252)** |  |
| --- | --- | --- | --- | --- |
|  |  |  |  |  |
|  |  |  |  |  |
|  |  |  |  |  |
| **Age** | **<50** | 27(26.0) | 87(34.5) |  |
|  | **≥50** | 77(74.0) | 165(65.5) |  |
| **ER** | **Positive** | 67(64.4) | 140(55.6) |  |
|  | **Negative** | 34(32.7) | 110(43.7) |  |
|  | **Other** | 3(2.9) | 2(0.8) |  |
| **PR** | **Positive** | NA | 126(50.0) |  |
|  | **Negative** |  | 124(49.2) |  |
|  | **Other** |  | 2(0.8) |  |
| **HER-2** | **Positive** | NA | 26(10.3) |  |
|  | **Negative** |  | 207(82.1) |  |
|  | **Other** |  | 19(7.5) |  |
| **pT** | **T1** | 18(17.3) | 57(22.6) |  |
|  | **T2** | 83(79.8) | 121(48.0) |  |
|  | **T3** | 3(2.9) | 66(26.2) |  |
|  | **Other** | 0(0) | 8(3.2) |  |
| **pN** | **Positive** | 59(56.7) | 133(52.8) |  |
|  | **Negative** | 45(43.3) | 116(46.0) |  |
|  | **Other** | 0(0) | 3(1.2) |  |

**Table S1.** The clinicopathological characteristics of two external validation cohorts.

Abbreviation: ER: Estrogen receptor; PR: Progesterone receptor; HER-2: Human epidermal growth factor receptor-2; pT: pathologically diagnosed tumor size; pN: pathologically diagnosed lymph node status.
